# Supplementary material for: Acceptance and compliance with micronutrient powder (MNP) among children aged 6–23 months in northern Nigeria
Source: PLOS Glob Public Health. 2022 Oct 17;2(10):e0000961. doi: 10.1371/journal.pgph.0000961 (PMC10022258; doi:10.1371/journal.pgph.0000961)
Supplement: S1 Dataset — (ZIP) [file pgph.0000961.s008.zip › S8_Dataset/Spot Checks.zip!/Spot Checks/11072015_A_GU_PM_2_SC a.pdf]

SPOT CHECK RECORDING FORM

State: Adamawa **LGA: ____Song____ Community: _Gombawa_**

Name of caregiver (female): Aishatu Adamu

Name or caregiver (male): ………………………

Sex of index child: _Male___ Age of index child (in months): _ 20 months__________

Spot Check Information

1. **Date of MNP distribution to household** (circle option below based on LGA name above)**:**

   - **Kebbi:** BirninKebbi&Jega LGA: **June 16**
   - **Kebbi:** Danko-wasagu: **June 17**
   - **Adamawa:**Song, Ganye: **June 18**
   - **Adamawa:**Guyuk **June 19**
   - **Adamawa:**Guyuk **June 24**

2. **Date of Spot Check Observation: ____11/7/2015_____**

3. **Number of sachets remaining**(Ask the caregiver to show you and count the specific number of sachets remaining)**:**

   32

4. **Number of sachets that <u>should remain</u>using one MNP sachet per day** (Analyst only)**:**

   39

5. **Number of sachets remaining / number of sachets should be remaining**(Analyst only)**:**

_____32____ / ___39______ = __82.1__ %

Probes

Question 1. You have **XX** MNP sachets remaining. Explain how easy or difficult it was to use the MNP product on a daily basis for your child 6 – 23 months? (Circle one answer)

Very Difficult **Difficult** **Easy** **Very Easy**

Question 2. Describe any specific challenges that you faced that made the MNP difficult to use on a daily basis?

The child does not like to drink the MNP. I have to force him. I think he does not like the taste and flavor.

Question 3. Describe specific aspects of the MNP product that you or your child enjoyed that made it easier to use on a daily basis?
To be honest with you the boy has seriously improved in his eating habit. He eat almost everything he sees around and the first few days I started given him the powder I noticed he added serious weight but later the weight dropped but he is doing fine and no problem at all.

Question 4. What questions do you have about the MNP based on your experience using it?

- I want to know the exact importance of this powder (MNP) to kids.

Question 5. What specific recommendations do you have that we can do as a program to make the MNP easier to use on a daily basis?

There should be regular supervision by supervisors who can monitor and ensure compliance. There should be some form of rewards to caregivers who use it very well and complete the dose as required.
